# Potential Physiological and Cellular Mechanisms of Exercise That Decrease the Risk of Severe Complications and Mortality Following SARS-CoV-2 Infection

**DOI:** 10.3390/sports9090121

**Published:** 2021-08-31

**Authors:** Johan Jakobsson, Ian Cotgreave, Maria Furberg, Niklas Arnberg, Michael Svensson

**Affiliations:** 1 Section of Sports Medicine, Department of Community Medicine and Rehabilitation, Umeå University, 901 87 Umeå, Sweden; michael.svensson@umu.se; 2 Division of Biomaterials and Health, Department of Pharmaceutical and Chemical Safety, Research Institutes of Sweden, 151 36 Södertälje, Sweden; ian.cotgreave@ri.se; 3 Department of Clinical Microbiology, Umeå University, 901 87 Umeå, Sweden; maria.furberg@umu.se (M.F.); niklas.arnberg@umu.se (N.A.)

**Keywords:** virus, immunology, physical activity, exercise, health, COVID-19, SARS-CoV-2, obesity

## Abstract

The coronavirus disease 2019 (COVID-19) pandemic caused by severe acute respiratory syndrome coronavirus 2 (SARS-CoV-2) has unmasked mankind’s vulnerability to biological threats. Although higher age is a major risk factor for disease severity in COVID-19, several predisposing risk factors for mortality are related to low cardiorespiratory and metabolic fitness, including obesity, cardiovascular disease, diabetes, and hypertension. Reaching physical activity (PA) guideline goals contribute to protect against numerous immune and inflammatory disorders, in addition to multi-morbidities and mortality. Elevated levels of cardiorespiratory fitness, being non-obese, and regular PA improves immunological function, mitigating sustained low-grade systemic inflammation and age-related deterioration of the immune system, or immunosenescence. Regular PA and being non-obese also improve the antibody response to vaccination. In this review, we highlight potential physiological, cellular, and molecular mechanisms that are affected by regular PA, increase the host antiviral defense, and may determine the course and outcome of COVID-19. Not only are the immune system and regular PA in relation to COVID-19 discussed, but also the cardiovascular, respiratory, renal, and hormonal systems, as well as skeletal muscle, epigenetics, and mitochondrial function.

## 1. Introduction

Coronavirus disease 2019 (COVID-19), which is caused by severe acute respiratory syndrome coronavirus 2 (SARS-CoV-2), rapidly increased to pandemic magnitude during the first quarter of 2020 [1]. The severity of COVID-19 appears to be a net consequence of the combination of SARS-CoV-2 infection and the person’s cardiorespiratory and metabolic fitness, age, sex, and ethnicity [2,3,4,5,6]. Some individuals who become infected with SARS-CoV-2 present with mild or no symptoms of illness, whereas others become critically ill and dependent on intensive care with a poor prognosis [7].

The physiological and cellular factors that interact and determine an individual’s vulnerability to SARS-CoV-2 remain to be determined. Research has highlighted several predisposing risk factors for mortality in COVID-19, many of which are related to low cardiorespiratory and metabolic fitness, including obesity, cardiovascular disease (CVD), diabetes [2,4,8,9,10], and metabolic syndrome (MetS) [11]. It is reasonable to assume that a physically active lifestyle results in physiological and molecular stress-induced adaptations in all tissues affected by exercise, which may be protective following SARS-CoV-2 infection, in contrast to the sedentary lifestyle associated with obesity [12].

The objective of this narrative review is to highlight potential physiological, cellular, and molecular mechanisms affected by regular physical activity (PA) and exercise that may regulate antiviral defenses and determine the course and outcome of COVID-19. First, we aim to explain the deterioration of fitness due to physical inactivity and sedentariness. Second, the biology of SARS-CoV-2 is briefly reviewed. Finally, we aim to illuminate the physiological and cellular effects of exercise on fitness and the potential of exercise as a protective measure against severe complications induced by SARS-CoV-2 infection. In addition to the immune system, also the cardiovascular, respiratory, renal, and hormonal systems, as well as skeletal muscle, epigenetics, and mitochondrial function in relation to regular PA and antiviral defense is discussed in novel aspects.

## 2. Definitions

PA is defined as any bodily movement generated by the contraction of skeletal muscles that raises energy expenditure above resting metabolic rate [13]. Exercise is a subcategory of PA that is planned and structured, favoring physical fitness. The World Health Organization (WHO) recommends either 150–300 min of moderate-intensity (3–6 metabolic equivalents, 1 metabolic equivalent = 3.5 mL of O_2_ per kg of body weight per minute) or 75–150 min of vigorous-intensity (>6 metabolic equivalents) PA per week for adults [14]. To confer additional health benefits, 300 min of moderate-intensity or 150 min of vigorous-intensity PA per week is suggested. Being physically active entails fulfilling these guidelines and not fulfilling them is considered being physically inactive [15]. Sedentary behavior is defined as any waking behavior with an energy expenditure ≤ 1.5 metabolic equivalents while sitting or lying. Consequently, a person can be physically active and still be classified as sedentary.

## 3. Fitness and Health Status, Immunity, and Infection

A sedentary lifestyle with obesity or MetS increases the risk of severe disease due to viral infections [16,17]. In contrast, PA elicits protective effects against both bacterial and viral infections [18,19,20,21]. How this knowledge can be extrapolated to COVID-19 is yet to be determined. However, emerging data indicate that obesity, diabetes, and MetS are some of the major risk factors associated with COVID-19 severity and mortality [4,8,22,23,24]. Although obesity is closely associated with all features of MetS, including abdominal adiposity, hypertension, dyslipidemia, and insulin resistance [25,26], PA and fair cardiorespiratory fitness are significant factors that counteract MetS, even in obese individuals [27]. Risk factors linked to obesity are more or less treatable with exercise [28], and epidemiological research has clearly shown that regular PA decreases the risk of comorbidities and all-cause mortality [29,30]. Common to several of the aforementioned risk factors and comorbidities are sustained low-grade systemic inflammation, characterized by increased levels of proinflammatory cytokines and disturbed regulation of fibrinolysis [31]. In this context, PA and exercise, or the lack thereof, play an important role in wide-ranging effects on brain, cardiorespiratory, metabolic, endocrine, muscular, and immunological functions.

## 4. Deterioration of Fitness Status during a Short Period of Physical Inactivity

Lockdowns and restrictions have affected the majority of humanity during the COVID-19 pandemic. Protective measures aiming to slow the spread of COVID-19 have affected the possibilities for PA and exercise according to data retrieved from activity trackers and mobile phones from all over the world [32,33]. These temporary restrictions have negatively affected the already ongoing crises of physical inactivity and obesity frequently addressed during the COVID-19 pandemic [34,35,36]. Longitudinal studies have shown that, in some cohorts, COVID-19 lockdowns led to an increase in body weight, snacking, and the consumption of ultra-processed foods, whereas activity decreased [37].

Physical inactivity and sedentary time are associated with multiple chronic diseases and premature mortality and contribute to a major economic burden worldwide [38]. Physical inactivity has been indicated to be the cause of 9% of deaths worldwide [39]. Physical inactivity plays a role, independent of other factors, in lowering cardiovascular and muscular fitness and, thus, the age of onset of chronic disease, decreasing the quality of life and shortening the health span [40]. Total sitting time, independent of PA, is associated with a higher risk of several major chronic diseases and all-cause mortality [41]. Physical inactivity and sedentary time are also substantial predictors of hospitalization and have been described as the central driver of mortality risk in longitudinal studies [42]. In contrast, increased PA at any intensity, of any duration, and less time spent being sedentary are associated with a reduced risk of premature mortality [30]. In terms of PA, doing something is better than doing nothing [29,30]; although more is better, a small dose of PA corresponding to 75 min per week, below current recommendations, substantially reduces mortality [43].

Changes in PA patterns are quickly reflected in human physiology. This is exemplified by the effects of 2 weeks of a reduced step count, which deteriorated multiorgan insulin sensitivity and muscle mass, and increased central and liver adiposity and dyslipidemia [44,45]. In the short term, prolonged sitting time negatively impacts metabolic markers, which is attenuated by PA [46]. Notably, from the perspective of COVID-19, physical inactivity per se is associated with low-grade systemic inflammation [47], which is further exacerbated by obesity [48], abdominal obesity in particular [49]. Undoubtedly, this is a major concern given restrictions, general mobility, and PA during the COVID-19 pandemic.

Accordingly, physical inactivity and a sedentary lifestyle are linked to an increase in all-cause mortality, and just a few days of being sedentary is sufficient to induce negative effects on metabolism, cardiorespiratory fitness, and skeletal muscle fitness.

## 5. SARS-CoV-2 and COVID-19

**Figure 1:**
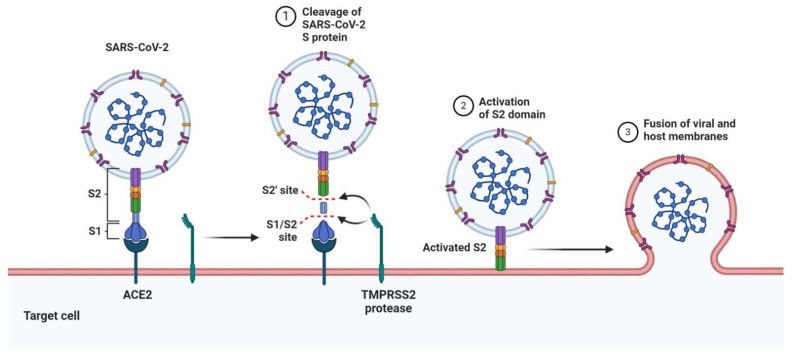
How SARS-CoV-2 can trigger the cytokine storm leading to severe COVID-19. The virus infects lung epithelial cells and is detected by resident macrophages, which trigger the production of interferons and cytokines. When more immune cells are attracted, more cytokines are produced, leading to a cytokine storm. Cell damage can occur via the formation of fibrin and weakened blood vessels, leading to fluid accumulation in the alveoli, respiratory failure, and general deterioration of organ function.

SARS-CoV-2 belongs to the *Coronaviridae* family within the order of *Nidovirales*. It transmits between humans mainly through droplets, but also via contact and/or aerosols [50]. The virus mainly targets the respiratory tract, but in some patients with severe illness, the virus disseminates to other organs, including the heart, liver, brain, kidneys [51], and intestine [52]. Infectious virions are equipped with a positive sense, single-stranded RNA genome that is unusually large compared to other RNA viruses. The genome encodes several non-structural and structural proteins, including the receptor-binding spike (S) protein, membrane (M) protein, envelope (E) protein, and a nucleocapsid (N) protein [53]. The cell and tissue tropism appear to be largely, but not entirely, dependent on the expression of the entry receptor angiotensin-converting enzyme 2 (ACE2), as well as cellular proteases, including TMPRSS2 [54]. Neutralizing antibodies mainly target the S protein, which contains a relatively high level of N-linked glycans compared to receptor-binding viral glycoproteins of other viruses [55]. These glycans partially protect the S protein from neutralizing antibodies. To interact with the ACE2 receptor, proteases, including TMPRSS2, cleave and activate the S protein, which undergoes a conformational change, resulting in exposure of the receptor-binding domain and facilitating a high-affinity interaction with ACE2 (Figure 1) [52,54,56,57].

Recent studies suggest that heparan sulfate [58] and neuropilin 1 [59] are additional host factors that can be used for attachment and cell entry by SARS-CoV-2 in addition to ACE2 and TMPRSS2, which together contribute to and regulate SARS-CoV-2 infection and tissue-tropism. Infection also activates innate immunity, which consumes additional energy. After cell entry, SARS-CoV-2 downregulates the expression of ACE2 [60]. This contributes to reduced uptake of more virus, but also inflammation and blood flow, potentially leading to multiorgan failure due to the overactivation of angiotensin (Ang) II [61]. Of significant interest here is the consequence of the physiological-cellular Ang II-ACE-ACE2 interaction following SARS-CoV-2 infection from the perspective of exercise versus MetS.

**Figure 2:**
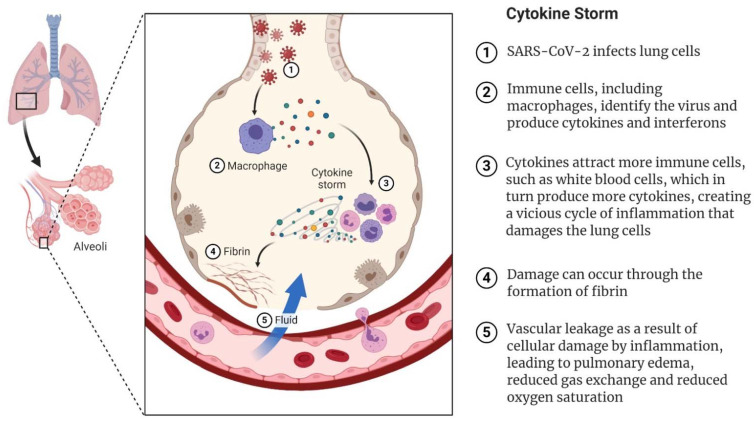
Mechanism of SARS-CoV-2 viral entry. Receptor-binding spike (S) protein engages with the host ACE2 receptor. TMPRSS2 cleaves and activates the S protein, which undergoes a conformational change, facilitating interaction with the host ACE2, driving the fusion of viral and host membranes. Recent studies also suggest that the S protein interacts with cellular heparan sulfate adjacent to the ACE2-binding site (not depicted).

COVID-19 mostly affects the respiratory system, although multiple extrapulmonary manifestations are common [62]. The initial infection of epithelial cells and the sequential pulmonary capillary endothelial cell infection prompt an influx of inflammatory cells, causing endothelialitis, alveolar wall thickening, and hyaline membrane formation, which are visualized at autopsy [63,64]. Interstitial infiltrates of leukocytes and edema create the typical ground-glass opacities seen on computer tomography. Ultimately, pneumonia vascular leakage and fluid accumulation lead to pulmonary edema, respiratory failure, hypoxia, and hypercapnia (Figure 2). Some critically ill patients develop fulminant acute respiratory distress syndrome (ARDS), septic shock, or multiorgan failure, and respiratory failure is the leading cause of death in patients with COVID-19 [65,66].

Pneumonia caused by SARS-CoV-2 is often accompanied by increased levels of C reactive protein (CRP), equivalent to those seen in bacterial pneumonia, and the secretion of hyaluronan, which affects oxygenation [67]. Some patients develop a hyperinflammatory syndrome and coagulopathy with a prothrombotic state. Thrombotic complications include both arterial and venous manifestations [62]. Critically ill patients have very high levels of inflammatory markers, such as CRP, erythrocyte sedimentation rate, fibrinogen, ferritin, interleukin (IL)-1, IL-6, and D-dimer. Leukocytosis is rarely seen, and lymphocytopenia is common. High levels of CRP and D-dimer and low lymphocyte counts are associated with poorer prognosis and death [68,69]. Disseminated intravasal coagulation (DIC) is a frequent occurrence [70]. However, COVID-19-associated hypercoagulopathy is dominated by thrombosis, whereas bleeding is the most prominent feature of DIC. The presence of endotheliopathy has been reported in COVID-19 and is likely associated with critical illness and death [62]. A subgroup of patients develops a cytokine storm syndrome, with unrestrained release of cytokines and devastating consequences for the host (Figure 2). Cytokine storm and hypercoagulopathy lead to destruction of the lungs, multiorgan failure, and thrombotic tendencies, with micro-thrombosis of the lungs further exacerbating lung tissue destruction. Immune-mediated lung injury (e.g., DIC and ARDS) is associated with adverse outcomes in patients with COVID-19 [63]. The hyperinflammatory response to the infection may be responsible for many of the commonly seen complications of the disease, such as lung damage, kidney failure, cardiovascular complications, neurological problems, and liver damage [71].

Redox regulators, mitochondrial factors, and non-coding RNAs are host factors affected by exercise and may dictate the outcome of SARS-CoV-2 infection [72]. PA and exercise induce several lines of stress, followed by adaptations if the exercise is repeated over time [28]. The adaptations are specific to the type, volume, and intensity of training and include physiological and cellular adaptations in many organs [73,74,75,76]. Some adaptive responses to stress following exercise include neuromuscular, respiratory, cardiovascular, hormonal, immunological, and cellular adaptations that improve or maintain factors that affect endurance, strength, and mobility, as well as health. In this context, the cellular and physiological mechanisms that determine the outcome of COVID-19, the effects of PA, and fitness state are of interest when examining differences in the impact of the virus in young vs. elderly, and in men vs. women as noted in epidemiological studies [4].

## 6. Effects of Physical Activity and Exercise on Human Biology in View of COVID-19

Epidemiological studies have shown that regular PA reduces the incidence of bacterial and viral infection across an individual’s lifespan and decreases the mortality and incidence of influenza and pneumonia [77,78]. Prospective studies have consistently shown that elevated levels of cardiorespiratory fitness and regular PA improve immunological function, confirming the association. Regarding COVID-19 and physical fitness, a few studies have shown protective effects of higher physical fitness (measured as cardiorespiratory fitness (VO_2_max), muscle strength, sport participation, walking pace or attending to PA guidelines). One study reported that maximal exercise capacity is independently and inversely associated with the likelihood of hospitalization due to COVID-19 [79]. Although more studies are warranted, cardiorespiratory fitness was argued to directly reflect the integrated function of multiple organ systems and an individual’s ability to tolerate cardiopulmonary stress. Another physiological rationale is the enhanced immune function with moderate PA. Another study showed that people over 50 years of age who engage in PA twice a week or more often have a lower risk of COVID-19 hospitalization, with the protective effect explained by muscular strength [80]. Others have found that regular sports participation, regardless of sex and age, positively affects the clinical outcome of hospitalized COVID-19 patients [81]. One study on self-reported walking pace, a simple measure of physical fitness, reported that slow walkers have almost twice the risk of severe and lethal COVID-19 compared to brisk walkers [5]. A prospective observational study in hospitalized patients with COVID-19 that assessed muscle strength on admission observed that the length of hospital stay was significantly shorter among patients in the highest tertile of strength [82]. Recently, a study investigating a cohort of 76,395 South Korean adults found that adults engaging in the recommended levels of PA had a decreased likelihood of SARS-CoV-2 infection, severe COVID-19 illness and COVID-19 related death [83]. In the aforementioned study, the authors argue that enhanced immunosurveillance, delayed onset of immunosenescence and reduced systemic inflammation, all to be discussed here, as possible explanations as immunological effects decreasing the risk of infection and severity due to COVID-19.

### 6.1. Adipose Tissue, Obesity, MetS, and Endocrine Effects in Relation to COVID-19 and the Effects of Exercise

Several of the detrimental effects of aging and low-grade systemic inflammation (specifically immunosenescence and inflammaging discussed in Section 6.2) go hand-in-hand with the effects of obesity, MetS, and physical inactivity [21]. Although obesity and MetS are risk factors for cardiovascular mortality [84], they also lead to a decrease in immune function [85], similar to immunosenescence. Regarding COVID-19, MetS is associated with increased mortality in SARS-CoV [86,87] and MERS-CoV [88,89] infection. Obesity was also determined to be a risk factor for the 2009 H1N1 pandemic [90].

Expansion of the adipose tissue (AT) mass during obesity is a key factor in increased systemic inflammation. The obese inflammatory state is characterized by elevated levels of TNF-α, IL-1β, IL-6, IL-17, IL-22, and CRP [91,92,93], which is also seen in physically inactive and sedentary individuals [47,49,94]. Reduced levels of anti-inflammatory and organ-protective adipokines (cytokines secreted by AT) are seen in obesity [95]. Pro-inflammatory Toll-like receptors (TLRs) are also overexpressed in obese subjects [96] as discussed in Section 6.2. Accumulation of AT, especially abdominal AT, leads to increased infiltration by pro-inflammatory M1-type macrophages, reducing the presence of anti-inflammatory M2 macrophages [91,92,97]. In turn, M1 macrophages release pro-inflammatory adipokines, including TNF-α, IL-1B, and IL-6. This shift towards M1 macrophages plays a central role in the systemic inflammation induced by obesity [98]. Some have called the baseline inflammatory obese state a barrier to the induction of a robust antiviral response, allowing for increased viral shedding and transmission [17]. Many argue that AT is a reservoir for extensive viral spread and increased shedding, not least of all due to the pro-inflammatory and immunocompromised state described below. This is also argued by Ryan and Caplice [99], as AT-resident cells are a proven target for multiple viruses: influenza A, SARS-CoV, adenovirus, and HIV target macrophages; SARS-CoV and HIV target lymphocytes; and H1N1, influenza A, and adenovirus target adipocytes.

Similar to other coronaviruses, SARS-CoV-2 activates the NACHT-, LRR-, and pyrin domain-containing 3 (NLRP3) inflammasome, increasing the production of pro-inflammatory cytokines [100,101,102,103]. As reviewed by Freeman and Swartz [104], NLRP3 has a known role in hyperinflammatory ARDS, severe MERS-COV, and SARS-CoV infection, and initial studies have indicated its involvement in severe COVID-19 [105]. Obesity, as well as aging, triggers activation of the NLRP3 inflammasome [106,107]. Thus, age-induced (inflammaging) and obesity-induced NLRP3 hyperactivity could predispose patients to the cytokine storm often seen in severe COVID-19 [106]. Thus, several NLRP3 inhibitors are in pre-clinical or clinical trials for the treatment of COVID-19, as summarized by van den Berg and te Velde [108]. Overall, obese individuals have been shown to be more susceptible to other infections, with more intense cytokine storms due to obesity-induced systemic inflammation [109].

In obese individuals, the antibody response to influenza vaccine is impaired [110]. Vaccinated obese adults have twice the risk of influenza compared to non-obese vaccinated adults [110]. Consequently, the primary means of preventing influenza, and speculatively COVID-19, could be weakened due to obesity. This is troublesome given the significant increase in obesity worldwide in the last few decades. An initial study showed that central obesity, as well as hypertension and dyslipidemia, is associated with lower antibody titers 1–4 weeks after the second inoculation with a COVID-19 mRNA vaccine [111]. Another study showed that higher BMI is associated with lower antibody titers in Italian health workers [112]. Furthermore, in obese individuals, the duration of influenza A virus shedding is prolonged [113], which could increase viral transmission. This has also been seen with SARS-CoV-2; obese patients require longer hospital stays and longer duration (6 days) to obtain a negative oropharyngeal or nasal swab [114].

Obesity and physical inactivity may both lead to endocrine abnormalities. Due to increased activity of the hypothalamic-pituitary-adrenal (HPA) axis, individuals with abdominal obesity have increased secretion of cortisol [115]. In addition to the adrenal cortex, cortisol production by adipocytes is also elevated in hyperinsulemic conditions [116]. Excess cortisol is, in turn, associated with increased abdominal adiposity and several cardiovascular sequelae, including, but not limited to, hypertonia, hyperglycemia, insulin resistance, dyslipidemia, and MetS [117]. Consequently, increased levels of cortisol induced by obesity and physical inactivity increase the risk of several comorbidities and risk factors for severe COVID-19 [4,24,118]. Contracting skeletal muscles release myokines that exert endocrine effects on abdominal adiposity, whereas other myokines have local paracrine effects in signaling pathways involved in fat oxidation [119]. Thus, physical inactivity leads to a metabolic decline, whereas regular PA allows for weight maintenance and/or the reduction of abdominal obesity and induction of an anti-inflammatory environment.

As ACE2 expression is a key factor for SARS-CoV-2 entry into host cells, it is reasonable to assume that low levels of ACE2 would protect against infection, viral amplification, and ultimately a lethal outcome in COVID-19. However, according to a recent model of viral dynamics and gene expression of ACE2 in rats and humans, there is a strong negative correlation between ACE2 and lethality [120]. This hypothesis agrees with research demonstrating that the ACE2 receptor is expressed at lower levels in men than in women, and decreases with age, inflammatory-related comorbidity, and with reduced testosterone and estrogen levels [121].

Regarding sex hormones, PA, and immunity, men with subnormal testosterone levels have higher concentrations of pro-inflammatory cytokines (IL-1β, IL-2, and TNFα) [122,123], which can be counteracted by testosterone supplementation [124]. In this regard, exercise has been shown to attenuate the age-related decline in testosterone in sedentary males [125,126]. Although exercise could diminish the age-related decline in testosterone, it seems to have no significant effect on basal levels in middle-aged men [127]. Testosterone is generally thought to be somewhat immunosuppressive, whereas estrogen is a natural enhancer of immunity [128,129]. In females, estrogen increases humoral immunity and has been shown to be antiviral, protecting against several viruses, including influenza A, HIV, Ebola, hepatitis C, and human cytomegalovirus [130]. This protection is due to several mechanisms, including increased viral-specific T cells (VSTs) in the lungs [131]. In postmenopausal females, exercise can increase estrogen levels, even in sedentary females. A small number of studies have examined the role of testosterone in COVID-19. In contrast to the immunosuppressive reputation of testosterone, some studies have found that low pre-infection levels of testosterone, or low levels of testosterone during infection, are related to worse COVID-19 outcomes [132,133]. However, these were preliminary studies and not rigorous randomized controlled trials. Notably, testosterone levels decrease during infection [134], making the association of lower levels and worse outcome a self-fulfilling prophecy. In addition, lower levels of testosterone often come with comorbid conditions and low metabolic fitness, including obesity, diabetes, and MetS [135]. Thus, testosterone possibly plays a role in driving ACE-2 and TMPRSS2 to increase viral entry [135], but no robust evidence yet shows that testosterone explains why men have a disproportionately high mortality rate in COVID-19.

### 6.2. The Immune System in Relation to COVID-19 and the Effects of Exercise

The host immunological response following infection with SARS-CoV-2 is crucial to the progression of COVID-19. An appropriate and well-regulated immune response with a balanced interaction between the innate and adaptive immune responses is key to controlling COVID-19 severity [136].

The immune system is influenced by PA and fitness status [78,91,119,137,138]. Age-associated deterioration of immune competency, immunosenescence, is a profound and multifaceted transformation that occurs gradually with age in humans [139,140,141,142,143,144,145,146,147], and is highly influenced by regular PA during one’s lifetime. With the vast majority of COVID-19 deaths occurring in the elderly and pathology data pointing to both immunosenescence and inflammaging/systemic inflammation as major risk factors for mortality [49,91,106,137,146,148,149,150,151], the effects of regular PA are of substantial interest.

**Figure 3:**
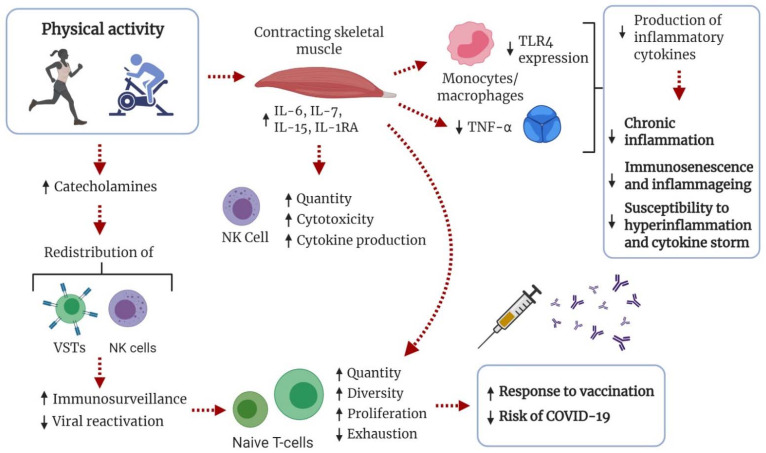
Schematic of immunological adaptations influenced by physical activity (PA) that could increase tolerance to COVID-19. In response to increased levels of catecholamines, viral-specific T cells (VSTs) and natural killer (NK) cells are redistributed in tissues. Immunosurveillance is increased and viral reactivation decreased, preventing T-cell senescence and increasing the number and diversity of naïve T cells. This is known to increase the response to influenza vaccination and decrease the risk of infection. During exercise, skeletal muscle releases myokines with anti-inflammatory effects. Importantly, production of tumor necrosis factor alpha (TNF-α) is inhibited. Regular PA can lead to downregulated expression of Toll-like receptors (TLRs) on monocytes and macrophages, leading to reduced production of pro-inflammatory cytokines.

TLRs are vital components in the innate immune system and important for the detection of pathogens and antiviral responses [152]. With advancing age, the TLR function is dysregulated, resulting in inappropriate TLR signaling and cytokine production [143,153]. Downregulated expression of TLRs on monocytes and macrophages, with reduced production of pro-inflammatory cytokines has been suggested as an anti-inflammatory effect of exercise [91,150,154,155]. In contrast, low metabolic fitness, such as obesity with insulin resistance or MetS, can increase the expression of TLR4 [156]. Exercise-induced alterations in TLR signaling, such as reduced TLR4 signaling [157], could attenuate the detrimental effects of low-grade systemic inflammation, improving general health and resiliency. Subsequently, this may attenuate the hyperactivation of monocyte-derived macrophages contributing to the cytokine storm and hyperinflammation in COVID-19 (Figure 3).

The SARS-CoV-2 S protein interacts with TLRs, especially TLR4 [158,159], leading to inflammatory responses. Therefore, in obese patients with increased expression of TLR4, the AT, which is already inflamed, makes a favorable environment for SARS-CoV-2-TLR4 interaction, exacerbating the pro-inflammatory cytokine production and increasing the severity of COVID-19 [160]. Treatment with TLR4 antagonists has been used successfully during other viral infections. As summarized elsewhere [161], TLR4 antagonists consistently reduce chemokine and cytokine production, mitigating disease symptoms. Excess activation of TLR4 plays a role in the pathogenesis of viral diseases. Several authors have suggested clinical trials to test TLR4 antagonists in the treatment of severe COVID-19 [162]. However, although no clinical trial data yet support its use, controlling the conditions related to an adverse outcome in COVID-19, such as being physically active, non-obese, and metabolically healthy, is essential.

Exercise and PA lead to an immunostimulatory catecholamine-mediated redistribution of NK cells and VSTs [78,146,149,163]. This increases immune surveillance and apoptosis of senescent T cells, and reduces reactivation of cytomegalovirus, Epstein-Barr virus, and varicella zoster virus. Consequently, the accumulation of senescent T cells is prevented, whereas the count and diversity of naïve T cells is maintained. Animal and human data support the notion that regular moderate-intensity PA improves viral defense effectiveness and decreases morbidity and mortality in viral infections and respiratory illnesses [21,78]. Importantly, increased PA has been shown to improve the immunological response to influenza vaccination [164,165]. Both acute and chronic exposure to exercise seem to enhance the immunological response to vaccination [166], but the optimal exercise regimen, dose-response relationships, and clinical importance are yet to be fully elucidated. Increased PA is also associated with better control of latent viral infections [167] and a reduced risk of pneumonia [18] and infectious disease mortality [19]. Emerging data show that the severity of the COVID-19 cytokine storm and its complications is associated with lymphopenia, with lower counts of and exhausted VSTs, CD4^+^ and CD8^+^ T cells, and NK cells [168,169,170]. High-intensity exercise has also been shown to enhance the effector profile of CD8^+^ T cells in mice [171] and obese humans [172]. Accordingly, the anti-immunosenescent effects of regular PA could be an important measure for maintaining resiliency against COVID-19 and other viral infections with advancing age (Figure 3).

In the context of leukocytes, physically active older adults have been shown to have increased telomerase activity and longer leukocyte telomere length compared to physically inactive peers [173,174]. This context is associated with better survival from sepsis and lower severity of ARDS [175]. Recently, shorter leukocyte telomere length was found to be associated with a higher risk of adverse COVID-19 outcomes, independent of other major risk factors, including chronological age [176,177]. As reviewed by Garatachea [178], habitual PA, especially aerobic exercise, as well as good cardiorespiratory fitness, is associated with longer leukocyte telomere length.

PA has anti-inflammatory effects important for attenuating not only inflammaging, but also general systemic inflammation as seen in the physically inactive and obese populations. Cytokines released by contracting skeletal muscles (“myokines”) have local and pleiotropic anti-inflammatory, immune-regulatory, and health-promoting effects [179]. With exercise bouts of sufficient load, IL-6 is exponentially released with exercise duration [180]. With increased levels of IL-6, there is a multiple-fold increase in circulating levels of anti-inflammatory cytokines IL-10 and IL-1ra). Importantly, exercise-induced release of IL-6 inhibits the production of the pro-inflammatory cytokine TNF-α [91,181], a master regulator of inflammatory cytokine production [182] highly involved in the COVID-19 cytokine storm (Figure 3). Although IL-6 is traditionally known as a pro-inflammatory cytokine vital in the initiation of the acute immunological response, research now supports IL-6 as a multifaceted cytokine capable of eliciting both pro- and anti-inflammatory effects depending on the context [183]. In addition, IL-7 [184] and IL-15 [185], which are both lymphocyte proliferative factors [186], are released during exercise, supporting the function and proliferation of immune cells, mainly naïve T cells. IL-15 is also essential for maintaining memory CD8^+^ T cells [187] and NK-cell activation, production, and cytotoxicity [188], making it an important factor in the stimulation of immune responses to infections. These transient exercise-induced changes lead to improved protection against sustained low-grade systemic inflammation and pro-inflammatory cytokines [180,181]. It is also important to mention that these anti-inflammatory effects are seen in both young and elderly people, and are even more evident in pathological conditions, such as obesity, CVD, and type II diabetes [47,180,189].

In summary, the connection between physical inactivity, obesity, and MetS with diminished viral defense is evident, creating an immunological case for increasing PA and reducing obesity and MetS in society [21]. Compared to an unfit, obese, and physically inactive individual, a fit, lean, and physically active individual has improved immune function and viral defenses. In the elderly, the anti-immunosenescent and anti-inflammatory effects of PA are of particular interest for increasing resiliency against COVID-19 and its cytokine storm.

### 6.3. The Cardiovascular System in Relation to COVID-19 and the Effects of Exercise

In addition to the hyperinflammatory state and coagulopathy, the pathophysiological features of the cardiovascular system observed in COVID-19 patients include detrimental effects on the heart and vascular compartment [190]. Regarding cardiac health, exercise training and endurance training, in particular, generate anatomical, functional [191], and metabolic [192] effects on the cardiovascular system that promote overall health and physical endurance.

Importantly, PA mitigates several of the cardiovascular and metabolic risk factors associated with COVID-19 severity, not the least of which is MetS [193]. In short, the therapeutic effects of PA improve coagulation and fibrinolytic homeostasis, endothelial function, and blood pressure regulation, increases blood volume and cardiac ejection fraction, and reduces myocardial oxygen demand and platelet aggregation [28]. This could decrease some of the major cardiovascular complications caused by COVID-19, including myocarditis, cardiogenic shock, and thromboembolism. Due to cellular adaptation to temporary exercise-induced stress, training has been proposed as a preconditioning strategy for cardioprotection against myocardial damage by ischemia/reperfusion [194,195].

Vascular immunopathology, coagulopathy, and thrombosis are well-recognized features of lung abnormalities in COVID-19 that correlate with mortality risk. An imbalance between the activators and inhibitors of the coagulation and fibrinolytic system is commonly seen in obesity, and an increased level of obesity positively correlates with COVID-19 severity and mortality [196]. Therefore, the disturbed hemostatic balance in overweight individuals, with increased coagulation and impaired fibrinolysis, prior to SARS-CoV-2-infection may increase the risk of severe COVID-19. Thus, regular exercise is a natural approach to either prevent or reduce inflammation, and preserve or restore hemostasis. Studies indicate a positive effect of exercise on the regulation of both coagulation and fibrinolysis [197]. This is strengthened by the fact that regular PA reduces all-cause and cardiovascular mortality [198] independent of the intensity of PA [199]. Additional adaptive responses to exercise are indicated by decreased red blood cell aggregation in obese men [200] and improved systolic blood pressure in men and women with pulmonary hypertension.

Due to the vascular adaptation to PA and exercise, the renin-angiotensin system (RAS), which includes ACE2, is of particular interest in the outcome of SARS-CoV-2 infection. ACE2 cleaves Ang II into Ang(1-7) [201]. The RAS is a complex endocrine system that plays an important function in controlling metabolic and cardiovascular homeostasis. In individuals with low fitness and/or cardiometabolic disease, there is an imbalance between the RAS axes, which have been described in depth by others [202]. In short, exercise shifts the balance of the RAS towards the protective ACE2/Ang(1-7)/proto-oncogene, G protein–coupled receptor (MAS) axis in relation to the ACE/Ang II/Angtiotension II receptor type I (AT1R) axis. The latter axis is often hyperactivated in individuals with diabetes, obesity, hypertension, and/or sustained low-grade systemic inflammation [203]. Similarly, increased Ang II is associated with vasculopathy and coagulopathy syndromes in COVID-19 patients [204]. In contrast, activation of the ACE2/Ang(1-7)/MAS axis is anti-inflammatory and vasodilatory, and has several protective effects on the cardiovascular and renal systems, such as anti-thrombolytic functions.

Manipulation of the RAS, balancing it towards the protective arm, is considered a potential therapy for COVID-19 [205]. Accordingly, it strengthens the significance of PA and exercise in improving fitness and the clinical outcome of COVID-19. This has also been argued by others [206,207], suggesting that higher levels of cardiorespiratory fitness may confer innate immune protection against COVID-19 by attenuating the cytokine storm by modulating the RAS and ACE2 activity. As reviewed by Magalhaes, et al. [208], the protective arm of the RAS plays a central role in COVID-19. Antihypertensive ACE inhibitors have successfully been used in the treatment of severe COVID-19 [205,209], and multiple clinical trials suggesting that Ang(1-7) can be used to treat COVID-19 are ongoing (see https://clinicaltrials.gov/ct2/show/NCT04332666, accessed on 5 June 2021, https://www.clinicaltrials.gov/ct2/show/NCT04401423, accessed on 5 June 2021 and https://clinicaltrials.gov/ct2/show/NCT04375124, accessed on 5 June 2021). Notably, ACE inhibitors act by modulating the RAS similar to the response to PA and exercise.

**Figure 4:**
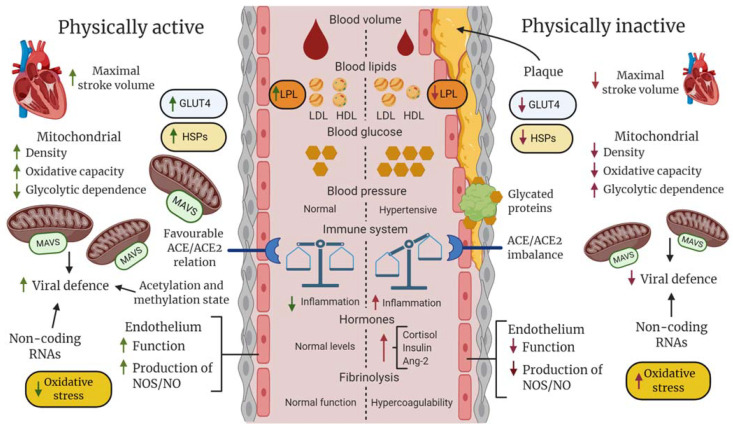
Hypothetical description of differences between fit (physically active and non-obese) and unfit (physically inactive and obese) individuals in regard to general vascular, blood, and cellular components that could collectively determine clinical outcome following SARS-CoV-2 infection. At the systemic level, regular physical activity (PA), such as exercise training, promotes hormonal homeostasis and improves maximal stroke and blood volume, enhancing blood perfusion and oxygen saturation of tissues. In an unfit state with hypertension, an imbalance between the innate and adaptive immune systems, increased platelet aggregation, dyslipidemia, and abnormally high glucose levels increase the risk of lipid deposition, protein glycation and clot formation, inflammatory responses in the vascular wall, and atherosclerosis, which decrease blood perfusion and oxygen saturation, potentially leading to an infarct in the worst-case scenario. At the cellular level, the temporary metabolic and mechanical stress induced during PA results in improved stress-defense (e.g., heat shock proteins [HSPs], antioxidants), improved mitochondrial capacity in aerobic adenosine triphosphate production and antiviral response (mitochondrial antiviral signaling protein [MAVS]), enhanced uptake and utilization of glucose and fatty acids via increased expression of glucose transporter type 4 (GLUT-4) and lipoprotein lipase (LPL), and improved endothelial functions, including the balance between ACE/ACE2, nitric oxide (NO) production, and endothelin release. Regular PA may also produce a favorable balance between epigenetic factors that facilitate the expression of genes encoding proteins that enhance general cellular functions and activate proteins and non-coding genes that inhibit amplification of SARS-CoV-2.

Collectively, exercise has a positive effect on the hemostatic balance and blood pressure, which may prevent high frictional resistance and shear stress on the vascular wall, coagulopathy, and thrombosis, reducing the risk of severe COVID-19. Furthermore, a well-regulated RAS and activated ACE2/Ang(1-7)/MAS axis induced by PA and exercise could be a significant factor in decreasing COVID-19 severity. These adaptations are summarized in Figure 4.

### 6.4. The Respiratory System in Relation to COVID-19 and the Effects of Exercise

From a pulmonary perspective, PA can increase the strength and endurance of the respiratory muscles [210]. Although it does not directly improve pulmonary function, it can make them more efficient and reduce the work of breathing. Concerning lung tissue damage and ARDS, PA can increase the diffusing capacity of the pulmonary alveoli. Endurance training is especially effective in this manner [211]. Highly fit, older adults have an increased diffusion capacity compared to less fit, age-matched peers [212]. In mice, aerobic exercise has been shown to attenuate acute lung injury by modulating the inflammatory and reactice oxygen species (ROS) balance [213,214,215].

Obesity also plays a role in respiratory dysfunction. It is associated with low lung volume and low respiratory muscle strength [216]. Being obese impairs respiratory mechanisms by increasing airway resistance and gas exchange [216]. Furthermore, obstructive sleep apnea (OSA) is more common in obese than normal-weight adults. However, obesity can also entail obesity hypoventilation syndrome (OHS), and some individuals suffer from both conditions. In OHS, obese individuals experience hypoventilation-induced hypoxia and hypercapnia regardless of the time of day, whereas OSA causes hypoventilation only during sleep [217]. Pre-existing OSA or OHS seriously limit respiratory function and, once affected by COVID-19, there is a minimum spare capacity. Accordingly, more mechanical ventilation is required in obese patients hospitalized with COVID-19 [218]. Abdominal adiposity can also obstruct the prone positioning necessary to avoid intubation in critically ill COVID-19 patients. Others have reported that abdominal adiposity and high intramuscular fat deposition are independent risk factors for critical care illness, mechanical ventilation, and death [219,220]. Therefore, regular exercise and being non-obese could improve respiratory resilience in COVID-19 due to the higher tolerance for pulmonary stress.

### 6.5. The Kidneys and Gastrointestinal System in Relation to COVID-19 and the Effects of Exercise

Reduced renal function is a risk factor for COVID-19 mortality [4]. Acute kidney injury (AKI) and chronic kidney disease (CKD) were evaluated in a recent meta-analysis, and both demonstrated a strong association with disease severity and mortality in COVID-19 compared to patients without CKD or AKI [221]. CKD and AKI are strongly interrelated with diabetes [222], and fasting blood glucose is an independent predictor of COVID-19 mortality in patients without a previous diabetes diagnosis [223]. Furthermore, ACE2 is highly expressed in the kidneys [224], offering a high possibility for SARS-CoV-2 infiltration and subsequent down-regulation of ACE2 by SARS-CoV-2. Thus, reduced expression of ACE2 in the kidneys, which reduces the ACE/ACE2 ratio, has been suggested as a possible cause of local vasoconstriction and inflammation, necrosis, and kidney damage in COVID-19 patients [225,226].

PA prevents many of the risk factors associated with the development of CKD, such as diabetic hypertension [227]. Some of the effects attributed to this include the preservation or improvement of insulin sensitivity, glucose tolerance, vascular endothelial function, hormonal balance, and altered adipocytokine profiles, which are all achievable through PA.

Although the direct effect of PA and exercise on the kidneys and renal function in humans remains unclear, PA prevents and reduces many of the aforementioned risk factors [227]. In this regard, the gene encoding protein Klotho is an attractive factor suggested as an anti-aging protein in the hallmark study by Kuro-o et al. [228]. Klotho is crucial for the performance of fibroblast growth factor (FGF) and involved in a multitude of regulatory roles, the metabolism of fatty acids and glucose, and anti-inflammatory and anti-oxidative actions [229]. In this context, Klotho supports and preserves blood vessel and kidney functions [230]. Klotho is abundantly expressed in the kidneys and brain, and modestly expressed in skeletal muscle [228]. The secreted, circulating form of Klotho (S-Klotho) is decreased with age, CKD, and CVD [231]. Interestingly, a 10- to 13-year prospective cohort of middle-aged males without any underlying disease demonstrated that low S-Klotho at baseline predicts an increased risk of developing high fasting plasma glucose (FPG) [232]. High FPG is a pre-diabetic state that also increases the risk of developing hypertension and kidney disease [233] and of death from COVID-19 [223].

A cohort study of master sprint athletes showed that they had better renal function, lower degree of inflammation, and higher Klotho levels than healthy but untrained age-matched peers [234]. This is in agreement with other studies that found higher S-Klotho levels in athletes compared to healthy controls [235] and an increased level with moderate-intensity exercise training [236]. Interestingly, a positive relationship has been shown between the S-Klotho level and the fat oxidation capacity at rest, as well as during exercise [237]. In rats, endurance training results in increased Klotho levels in the brain and kidneys, accompanied by an increased life span and attenuation of excessive ROS production [238]. In mice, the protein level of Klotho is acutely decreased in the skeletal muscle following one bout of exhaustive exercise, accompanied by a marked increase within 24 h compared to baseline [239]. The acute decrease in Klotho in the skeletal muscle may contribute to the increase in S-Klotho seen in studies of humans, which indicate a role of S-Klotho as a health-preserving myokine in the prevention of severe COVID-19. Furthermore, a marked acute increase in S-Klotho by exercise observed in endurance runners, but not in sprinters [240], suggests that the increase in S-Klotho could be dependent on aerobic fitness level and composition. A release of Klotho into the bloodstream from the skeletal muscle, despite relatively low expression per mass, could potentially account for a significant amount of S-Klotho, particularly as an effect of exercise training, because the muscle mass comprises approximately 30–40% of the total body mass. Consequently, muscle-derived Klotho may represent a novel myokine that may help explain some of the health and anti-aging effects of PA via anti-inflammatory, anti-oxidative effects and the preservation of vessel functions, which ultimately protect against severe COVID-19. Klotho is a promising candidate for predicting health status and as a biomarker for evaluating rehabilitation progress post-COVID-19.

Collectively, it is generally accepted that PA prevents the development of hypertension [28], insulin resistance [46,241], endothelial dysfunction [28], and systemic inflammation [91,150,151]. The regulatory factors underlying increased Klotho levels and improved ACE2/ACE ratio in the kidneys in relation to lifestyle factors, such as exercise, are yet to be discovered.

Gastrointestinal (GI) symptoms, such as diarrhea, nausea, and abdominal pain, are commonly seen in COVID-19 patients [242]. The high expression of ACE in the GI tract [243] may partly explain the vulnerability of the GI tract to SARS-CoV-2. Research on effects of PA and exercise on the GI tract is limited. However, regular PA seems to improve the microbiota composition and homeostasis of the immune system [244]. Intestinal dysbiosis and a leaky gut may contribute to the systemic inflammation generally seen in MetS. Clinical observations demonstrate that many COVID-19 patients have severe GI symptoms and detectable SARS-CoV-2 RNA in their stool, along with microbiome dysbiosis and inflammatory indicators [245]. A clinical study including patients with type 2 diabetes showed that 6 months of exercise, combined with caloric restriction, improved the intestinal microbiota composition and gut barrier function and reduced intestinal mycetes overgrowth and systemic inflammation [246]. Furthermore, a growing body of evidence indicates that obesity, MetS, intestinal dysbiosis, and asthma are closely associated. Interestingly, improved asthma control, lung function, and airway hyperresponsiveness are often seen after bariatric surgery, which also goes along with an improved gut microbial ecosystem and glycemic control [247]. Therefore, evidence indicates a close immunological link between the GI and respiratory tracts. At this point, a gut–lung axis has been proposed that affects the outcome of microbial infections and the progression of several diseases [248].

Whether exercise improves the mucosal dysbiosis in the lungs and intestines, thus preventing the development of severe COVID-19, remains to be elucidated. However, maintaining gut barrier function and controlling systemic inflammation by being metabolically healthy is advisable.

### 6.6. Skeletal Muscle in Relation to COVID-19 and the Effects of Exercise

The regular use of the skeletal muscles via PA and exercise training has fundamental effects at several physiological levels, including an acute increase in the utilization of fatty acids and glucose, followed by muscle adaptations that enhance the uptake of these energy components during exercise [249]. These adaptations to training are important for handling the challenge of the acute increase in blood glucose and lipids following a meal, preventing sustained vascular stress by these factors and, ultimately, CVD (Figure 4). The role of acute exercise and adaptation to training in relation to glucose and fat metabolism and the prevention of CVD and type 2 diabetes are reviewed in depth elsewhere [250,251,252].

Contrasting physiological, cellular, and molecular adaptations are developed through a lifestyle with or without regular PA. Regular PA, particularly recurrent exercise training, results in an improved capacity within the energy systems to meet the increased demand for adenosine triphosphate (ATP) during PA [253]. Exercise training also results in adaptations related to other challenges, such as mechanical, heat, and hormonal stress, increased levels of ROS [254], decreased blood and muscular pH and oxygen, the accumulation of metabolites, and changes in ion homeostasis.

Both endurance and resistance workouts [255] are accompanied by an acute increase in BP. Extremely high systolic and diastolic BP can occur during weightlifting, exemplified by a group mean value of 320/250 mmHg (highest 480/350 mmHg) during leg press with heavy resistance [256]. With this in mind, regular exercise has been shown to reduce the BP at rest in hypertensive subjects [257,258]. Furthermore, PA and exercise training improve hemodynamics and prevent vascular damage. Exercise-induced exposure to hydrostatic pressure and shear stress leads to adaptations that may prevent pro-inflammatory responses and vasculopathy following infection with SARS-CoV-2. These include increased expression of endothelial nitric oxide synthase (eNOS), which promotes vasodilatation via NO, increased capillarity, enhanced autonomic tone, and production of the vasoconstrictor endothelin-1 [259]. All of these may prevent pro-inflammatory responses and vasculopathy following infection with SARS-CoV-2. In obese rats, endurance training has been shown to modulate the cardiac RAS pathways, enhancing the ACE2 pathway over the ACE pathway [260]. Hypertensive rats subjected to endurance training have reduced blood pressure, decreased levels of the Ang II receptor AT1R, and increased levels of the Ang-(1-7) receptor MAS in brain tissue, with a parallel decrease in the production of ROS [261].

A potential adaptive response in humans to exercise training, such as reduced cell signaling in the Ang II pathway and increased activity of the ACE2-Ang-(1-7)-MAS pathway, may also enhance cellular energy metabolism in addition to increased defenses against ROS. In addition to the negative effects on the vascular system of the hyperactivated RAS, Ang II has been shown to suppress the phosphorylation of AMP-activated protein kinase (AMPK) in rat skeletal muscle [262]. This is an important finding, as AMPK is a central regulator of adaptation to exercise training, such as improved insulin sensitivity and glucose uptake [263]. As hyperglycemia is a risk factor for CVD and severe COVID-19 [223], the metabolic functions of the skeletal muscle are of major importance for health. Maintaining muscle mass and function with PA is crucial to sustaining insulin sensitivity and glucose disposal and protecting against comorbidities [264]. Dysfunctional skeletal muscle, as seen with chronic inactivity, aging, and tissue damage, is a precipitating cause in conditions such as type II diabetes, CVD, and cancer.

### 6.7. Epigenetics in Relation to COVID-19 and the Effects of Exercise

Epigenetic factors are modulated by exercise [265] and may play a protective role in the physiological and cellular responses to viral infection. Epigenetics includes factors that regulate gene expression through histone methylation and acetylation, DNA/RNA methylation, chromatin remodeling, and non-coding RNAs [266]. Recently, a role of microRNAs (miRNAs) was suggested in the regulation of translation processes involved in SARS-CoV-2 infection and its disease, COVID-19, by either blocking or degrading mRNA with subsequent knockdown or suppression of specifically related genes [267,268]. MiRNAs are non-coding RNAs that play a role in the regulation of gene expression, binding to target gene mRNAs and inducing mRNA degradation to inhibit protein synthesis [269]. A potential role of miRNA in lung diseases is exemplified by elevated levels of several sequences found in the plasma of patients with chronic obstructive pulmonary disease (COPD) [270] and may play a role in COVID-19. Interestingly, recent research identified 42 potential human antiviral miRNAs predicted to target SARS-CoV-2 [271]. Among these, circulatory miR-125a has been shown to increase in response to high-intensity exercise [272], as well as miR-23b in skeletal muscle following aerobic exercise, but without any concordant increases in plasma vesicular miR-23b [273,274,275,276]. The role of exercise-induced miRNAs in the obstruction of viral gene expression is an exciting field of research for the future. The miRNA signature in the cardiorespiratory system, skeletal muscle, kidneys, brain, blood vessels, and blood plasma may all respond to exercise and correlate with fitness status; thus, they may have a protective role in the defense against detrimental effects of SARS-CoV-2.

It could be hypothesized that the protective feature of regular PA and exercise training involves inhibition of host cell gene expression that would otherwise be “highjacked” by SARS-CoV-2 in the development of COVID-19. Exercise has been shown to induce significant epigenetic changes, regarding both the acetylation and methylation of histones [277,278]. Histone acetylation and methylation are reversible regulatory processes that facilitate and obstruct, respectively, gene expression [279]. However, epigenetic research in exercise biology is at an early stage and skewed heavily towards muscle function research. Nonetheless, in the context of vulnerable groups at higher risk of developing COVID-19, hypermethylation of DNA in the muscle has been observed with aging [280] and reversed in type 2 diabetes with training [280]. Increased histone acetylation in skeletal muscle has been shown following resistance training [281], as well as an increased frequency of genome-wide hypomethylation, which was preserved over a detraining period and further enhanced in response to re-training [282]. Changes in host-derived long non-coding RNAs (lncRNAs) were recently identified in the lung tissues of patients with COVID-19 [283]. LncRNAs have been suggested to play an important role in the regulation of immune responses and comorbidities [284]. Interestingly, lncRNAs have been shown to be differentially expressed in skeletal muscle between different training protocols [285]. However, the specific roles of lncRNAs in the host response to SARS-CoV-2, or in exercise biology, remain to be investigated.

Collectively, PA and exercise result in epigenetic changes (Figure 4) together with increased expression of genes linked to enhanced muscle functions [286,287,288,289]. Whether changes in the epigenetic signature seen in human skeletal muscle following training correspond to similar changes in the cardiovascular-respiratory system is yet to be determined, although this has been emphasized [290,291,292,293]. Although speculative, the aforementioned epigenetic changes could potentially be involved in increasing resiliency towards viral infection, including SARS-CoV-2 infection.

### 6.8. Mitochondrial Health in Relation to COVID-19 and the Effects of Exercise

The mitochondrion is the aerobic powerhouse that generates essential ATP in all cells that contain this organelle. Beyond the synthesis of ATP, the mitochondrion produces cofactors, lipids, and cell signaling factors and plays a role in steroid metabolism, calcium homeostasis, and apoptosis [294]. Furthermore, a vital role of the mitochondrion in antiviral defense has been discovered in recent years [295].

The quality and quantity of the mitochondrial mass within each cell are significant for the rate of ATP synthesis, substrate flexibility, stress responses, homeostasis, the cellular metabolic rate, health, and survival. Therefore, the mitochondrial capacity may be a crucial factor in organs infected by SARS-CoV-2. Related to risk factors associated with COVID-19, a reduction in PA results in reduced mitochondrial oxidative capacity and insulin signaling in human skeletal muscle [296]. Furthermore, hyperglycemia and overproduction of ROS by the mitochondria have been suggested to be a major trigger of the inflammation and vascular damage seen in type 2 diabetes [297]. Sustained low-grade systemic inflammation, which is commonly observed in individuals at high risk of COVID-19 [298], is associated with increased mitochondrial ROS production in the endothelium, as well as vascular dysfunction [299]. Therefore, pro-inflammatory cytokines and ROS likely produce a vicious cycle in COVID-19 patients [300]. In contrast to reduced PA, increased PA via endurance training has been shown to improve mitochondrial function and the lipid oxidation capacity in both lean and obese subjects [301], and to enhance the insulin sensitivity and glucose uptake [302]. It has been hypothesized [303], and now shown [304], that SARS-CoV-2 manipulates the mitochondrial metabolism. Findings from COVID-19 patients suggest that mitochondrial dysfunction drives a systemic immune response in COVID-19 pathogenesis. As suggested by Ajaz et al. [304] and others [305,306], maintenance of mitochondrial health and function is essential for an adequate innate immune system response to counteract the modulation by SARS-CoV-2

Mitochondrial biogenesis, the process that increases the mitochondrion density and aerobic oxidation capacity following exercise, is not restricted only to the skeletal muscle. This is exemplified by enhanced mitochondrial respiratory capacity, redox balance, abundance of manganese superoxide dismutase (MnSOD), and NO bioavailability in the arteries of mice following training [307]. Furthermore, exercise training in rats has been shown to increase the activity of antioxidative enzymes in several tissues, including the brain, liver, lungs, and muscle [308]. This would be expected to decrease the pro-oxidative tone of the intracellular milieu, favoring a more reduced and physiologically advantageous environment for carrying out normal cellular processes. In addition, the level of heat shock protein 70 (HSP70) has been shown to be upregulated in the lungs of rats after exercise training, which attenuates heat-induced acute pulmonary edema, inflammation, and ischemic and oxidative damage in the lungs [309]. Acute thermal stress measured during exercise, with a temperature of 38–40 °C in the core [310] and skeletal muscle and brain, is likely to be another factor that stimulates the expression of HSPs in many organs [311]. Related to the panorama of risk factors for COVID-19, an imbalance between extracellular HSP70 (eHSP70) and intracellular HSP70 (iHSP70) which is counteracted by exercise, has been proposed to play a role in type 2 diabetes, with eHSP being more pro-inflammatory and iHSP70 more anti-inflammatory and associated with better insulin sensitivity [312] (Figure 4). Individuals with chronic disease that is inflammatory in nature (obesity, type 2 diabetes, CVD) have a disturbed and suppressed anti-inflammatory heat shock response. Therefore, NF-kB and NLRP3 inflammasomes are activated, leading to a massive cytokine storm, predisposing these patients to severe COVID-19, as also discussed by Heck, et al. [313].

We could further speculate that a difference in vulnerability to COVID-19 between fit and unfit individuals is related to differences at the level of and interconnections between inflammatory exposure, cytoprotection, and mitochondrial capacity. In line with this speculation, the mitochondrial dysfunction generally seen with aging and in comorbidities related to metabolic syndrome and low-grade systemic inflammation has been highlighted in relation to the poor prognosis of COVID-19 [314]. It can also be speculated that regular exercise produces some anti-viral mechanism in host cells that inhibits the replication machinery of the invading SARS-CoV-2 and, thus, the pathogenesis of COVID-19. Here, the effect of exercise training on the mitochondrial antiviral-signaling protein (MAVS) and regulating factors is interesting, but not yet explored. As reviewed by Refolo et al. [295], the MAVS signalosome can rapidly induce the expression of hundreds of genes with antiviral properties in response to infection with an RNA virus. This is further discussed by Burtscher, Millet and Burtscher [306], who argued for mitochondrial integrity and, thus, enhanced MAVS. However, increased fatty acid metabolism could support viral replication in obese individuals. Several studies have demonstrated that intracellular lipid droplets interact with RNA viruses and can support their replication and the production of inflammatory mediators [315,316]. Therefore, increased mitochondrial function and lipid metabolism via regular PA and being non-obese may be other factors that affect the outcome of COVID-19 between fit and unfit individuals [305]. In addition, increased mitochondrial density (i.e., increased number and size of mitochondria) in all tissues affected by an increased metabolic rate because of exercise training may increase the density of MAVS, which enhances the anti-viral response following infection by SARS-CoV-2 (Figure 4). Lastly, enhanced mitochondrial function has been shown in blood platelets with exercise, in stroke patients [317], and in patients with heart failure [318], along with improved aerobic capacity. Thus, improved mitochondrial function in platelets after PA and exercise may reduce thrombogenesis and the risk of coagulopathy following SARS-CoV-2 infection.

## 7. Conclusions

The COVID-19 pandemic has unmasked mankind’s vulnerability to biological threats. Old age and obesity with one or more comorbidities are clearly defined risk factors for developing severe COVID-19. These risk factors are all associated with a sedentary lifestyle and low cardiorespiratory fitness. However, PA, such as exercise, generates cellular and physiological responses that support health and mobility and prevent comorbidities and mortality. Research on the effects of viral infections at the level of cellular and physiological mechanisms in relation to physical fitness is clearly lacking. Hopefully, these potential links will be explored in the future in light of the COVID-19 pandemic. Efforts must be made against the pandemic of obesity and physical inactivity to increase the average cardiorespiratory, metabolic, and immunological fitness of the population, thus increasing host antiviral defenses and the pandemic resistance of mankind. A decreased response to vaccination and prolonged virus shedding in obese individuals must also be stressed and further investigated in controlled studies. Research on exercise regimens, including modality, duration, and intensity, as well for increasing immunological fitness and the immunological response to vaccination, will benefit our society in future pandemics. The prevention and treatment of obesity and low physical fitness will likely improve mankind’s resilience against a global viral challenge, so aptly illustrated by the current COVID-19 pandemic.

## Data Availability

Not applicable.
